# Recovery Barrier Characterizations by Hospitalized Patients with Substance Use Disorders: Results from a Randomized Clinical Study on Inpatient Peer Recovery Coaching

**DOI:** 10.3390/ijerph21010093

**Published:** 2024-01-14

**Authors:** Kaileigh A. Byrne, Prerana J. Roth, Sam Cumby, Eli Goodwin, Kristin Herbert, William Michael Schmidt, Samantha Worth, Kyleigh Connolly, Onye Uzor, Brandi Eiff, Dominique Black

**Affiliations:** 1 Department of Psychology, Clemson University, Clemson, SC 29634, USA; brandi.eiff@kcl.ac.uk (B.E.);; 2 Greenville Memorial Hospital, Prisma Health, Greenville, SC 29605, USA; 3 Greenville Campus, University of South Carolina School of Medicine, Greenville, SC 29605, USA; scumby@email.sc.edu (S.C.); wms1@email.sc.edu (W.M.S.); sworth@email.sc.edu (S.W.);

**Keywords:** substance use disorder, peer recovery coaching, recovery capital

## Abstract

Patients hospitalized with medical complications from substance use disorder (SUD) encounter unique health problems that may complicate their recovery. Recovery barriers are not well understood in this population. The study objective is to characterize recovery barriers in this patient population. Participants (*n* = 96) in this six-month longitudinal study were randomized to a peer recovery coaching intervention or standard of care. The primary outcome measures were qualitative, open-ended questions addressing factors interfering with participants’ recovery. Data were analyzed using content analysis. Themes were identified a priori using past research on recovery capital domains; these seven barriers were (1) psychological health difficulties, (2) physical health challenges, (3) lack of social support, (4) insufficient treatment or recovery support to maintain sobriety, (5) environmental and housing concerns, (6) deficits in coping skills, and (7) lack of meaningful activities. At baseline, the most common recovery barriers were in the environment and housing (28.1%), psychological health (27.1%), and social support (22.9%) domains. At six-month follow-up, participants were asked to describe barriers they felt they had made improvement in over the last six months. The primary themes that participants reported improvements in were treatment and recovery support to maintain sobriety (52.1%), coping skills (35.4%), and social support (27.1%). Hospitalization and participation in a randomized controlled trial may be a turning point in which to address recovery barriers for patients hospitalized with complications from SUD.

## 1. Introduction

In 2020, 40.3 million people in the United States aged 12 or older (or 14.5%) were classified as having a substance use disorder (SUD) [1]. SUD care has historically focused on an acute care model that focuses on medical stabilization but largely fails to consider the individual barriers to recovery care patients face once they are discharged [2]. Indeed, many patients admitted for SUD or alcohol use disorder (AUD) face challenges with social determinants of health, such as housing, social support, and chronic pain, that complicate their recovery [3]. Thus, there is a need for innovative recovery approaches that provide better long-term health outcomes and mitigate recovery barriers post-discharge. One potential effective approach is the use of recovery coaching services. Peer recovery coaching offers a personalized recovery care service that is tailored to a patient’s specific needs [4,5,6]. However, there is limited research aimed at characterizing patients’ own perspective of their recovery barriers, particularly among patients hospitalized with medical complications from SUD. Peer recovery coaching in the hospital system could help mitigate an individual’s unique recovery challenges. Consequently, the objective of this study is to understand barriers to recovery and changes in these barriers over a six-month period.

Barriers to SUD recovery are multifaceted and compounding [7,8]. To overcome such barriers, the concept of recovery capital has been used to delineate specific domains that can predict recovery outcomes [9,10]. Recovery capital refers to internal and external assets that help support initiation and sustained recovery from AUDs and SUDs [11,12]. These assets include but are not limited to socioeconomic status, social connections, living environment, and access to care. Past research has outlined ten recovery capital areas or domains that are key to recovery: psychological health, physical health, community involvement, social support, meaningful activities, environment/safety, coping/life functioning, substance use and sobriety, risk-taking, and recovery experience [8]. Recovery barriers and recovery capital are inversely related: if an individual has poor recovery capital in a particular area, then it is likely that area represents a barrier to one’s SUD recovery.

Although approximately 40–60% of patients treated for a SUD experience relapse [13], improving one’s recovery capital has the potential to foster positive recovery outcomes. For example, greater recovery capital predicts substance use treatment completion and reduced substance use frequency following treatment [10,14]. Recovery capital has also been shown to be a protective factor for continued abstinence across stressful events [15]. It is possible that recovery capital enhances individuals’ ability to effectively cope with stressors or triggers [15]. Furthermore, greater recovery capital is also predictive of improved satisfaction with one’s life and lower perceived stress at one-year follow-up [16].

Individuals hospitalized with medical complications for SUD may be at particularly high risk for experiencing barriers to SUD recovery. Common medical complications include alcohol and related complications (e.g., delirium tremens, acute pancreatitis, alcoholic hepatitis), serious infections (e.g., endocarditis, osteomyelitis, sepsis), hypertensive crises, and overdose [17,18]. These patients may have high rates of against-medical-advice (AMA) discharge that endangers both their SUD and physical recovery [18,19]. Untreated withdrawal symptoms, intense cravings, uncontrolled pain, and feelings of stigma from medical providers increase the likelihood of AMA discharge [20,21]. Moreover, it is rare for hospitals to address patients’ SUDs during acute inpatient hospitalization [22,23]. The stress and physical toll of medical complications from SUD may add another barrier to these patients’ recovery, yet the root cause of the medical emergency—the SUD—is often not addressed.

Despite these negative outcomes, hospitalization represents a ‘reachable moment’ to initiate recovery in this population [18]. Over half of patients hospitalized with complications from SUD report an interest in reducing their substance use [24]. Furthermore, the hospitalization itself can prompt motivation for change. Patients undergo detoxification during their hospitalization and are faced with the salient fact that their medical condition could worsen if their substance use does not change [3]. Hospitalization can, therefore, be an ideal time to discuss recovery options and ways to mitigate recovery barriers with these patients. Indeed, previous research has shown that an inpatient addiction consult service can improve initiation and engagement in SUD treatment [18]. Relative to engagement rates at baseline, 6% more participants initiated treatment or recovery care following hospitalization only, while 22% more participants initiated care following hospitalization plus an addiction consult [18]. This finding suggests that the hospitalization experience can prompt initiation into treatment or recovery care, but addiction consultation has significant benefits over and above hospitalization alone. In the present study, all patients are counseled on the risks of substance use and are provided with addiction recovery support services through participation in a randomized clinical study. While this is not the same as an addiction consult service, it may nevertheless be beneficial in prompting recovery compared to hospitalization alone. Thus, this study seeks to understand changes in patients’ perceived recovery barriers following an acute inpatient hospitalization.

Few studies examining recovery barriers have utilized patient input and direct experiences. However, one qualitative study that investigated the experience of hospitalized patients with SUD showed that there are several key factors that can influence patients’ readiness to change [3]. A common theme was that hospitalization fails to address stressors and trauma that underlie urges to use, which made providers seem out of touch with the patients’ needs. Similarly, patients often felt stigmatized by providers [3]. Other qualitative research has echoed these themes; individuals in early recovery reported that limited connection and empathy from clinicians, lack of personalization to one’s needs, and poor understanding of patient struggles all pose obstacles to maintaining early recovery [25]. To improve recovery initiation, patients emphasized the significance of engaging with peers who have lived experience, as it offered valuable support and served as a model to demonstrate that recovery is achievable [3]. It is also important to consider changes in recovery barriers as patients progress from recovery initiation to early recovery to sustained recovery. Another qualitative study showed people’s priorities and needs shift at different stages of recovery, including factors such as employment, family and social relations, and housing [26], which highlights the need for consistent patient input to achieve sustained recovery. Consequently, the present study examines changes in patients’ perceived recovery barriers six months after initial hospitalization.

A key problem in facilitating recovery for SUD is poor access to treatment and recovery services. Peer recovery coaching may help bridge this gap in recovery care. Peer recovery coaches are individuals who highlight their own lived experience of recovery from SUD by providing peer-based mentoring, education, and support services to individuals with SUD [27]. Peer recovery coaches focus on immediate recovery needs and mobilization of personal, family, and community recovery capital; exhibit respect for diverse pathways and styles of recovery; and use their experiences as a helping instrument to emphasize a continuity of recovery support over time [28]. Preliminary evidence suggests that peer recovery coaching may help address the need for accessing care and has been shown to promote engagement in a full continuum of recovery care in a recent randomized controlled trial [29,30]. Collectively, this work shows that peer recovery coaching shows promise in addressing deficits in recovery capital.

The aims of the present investigation are twofold. The first aim is to assess patients’ perceptions of factors that hinder their recovery from SUD. Patients’ own accounts of factors that prevent them from starting and maintaining recovery are often overlooked in addiction research, and thus, the proposed investigation solicited SUD patients’ input. Given that increasing recovery capital is a critical factor in SUD recovery, we sought to use recovery capital domains as a framework to categorize patients’ responses. The second aim of this study was to characterize patients’ perceived improvement in overcoming recovery barriers six months after hospitalization. As an exploratory aim, we also sought to assess whether peer recovery coaching might mitigate certain perceived recovery barriers. To accomplish this, we assess responses from a study that compares a hospital-initiated peer recovery coaching service to the current standard of care (SOC) over a six-month period among patients hospitalized with SUD complications.

## 2. Materials and Methods

### 2.1. Study Design

The study design entailed a six-month prospective study in which participants were randomized to either the intervention or standard-of-care control using REDCap version 8.10.7 (Vanderbilt University, Nashville, TN, USA). As part of this study, participants completed both descriptive, open-ended questions about recovery barriers and quantitative measures of engagement, substance use frequency, and mental health. The consolidated criteria for reporting qualitative research (COREQ) was used to guide reporting for this study [31].

### 2.2. Study Setting

This study was performed at Prisma Health, Greenville Memorial Hospital (GMH), a large teaching hospital in South Carolina. GMH is a 746-bed regional referral center and the only 24 h Level I trauma center in the county. At the time of this study, this hospital did not have an inpatient addiction consultation service.

### 2.3. Participants

The target population was patients who were hospitalized due to complications from their SUD (e.g., severe alcohol withdrawal, pancreatitis, endocarditis, hepatitis, etc.). The target recruitment goal was 100 participants. The majority of patients were experiencing serious health problems as a result of long-term (M = 13.87 years, SD = 9.70) substance use. The study inclusion criteria were as follows: minimum of 18 years old, inpatient in the internal medicine or infectious disease services, identified by healthcare provider as having SUD due to alcohol, opioids, or methamphetamine. Participants were excluded from study participation if they were over 60 years of age; pregnant; a non-English speaker; unable to provide informed consent due to intubation, confusion, or related factors during hospitalization; or did not have a method of contact for follow-ups.

### 2.4. Study Conditions

#### 2.4.1. Peer Recovery Coaching Intervention

Two peer recovery coaches (both females in their 40–50s) delivered the intervention. Peer recovery coaches in this study were defined as Certified Peer Support Specialists (CPSS) who had been in recovery for at least two years. The intervention entailed a hospital-initiated bedside hospital visit from a peer recovery coach. After the initial bedside encounter, the peer recovery coach initiated contact with the participant a minimum of twice weekly for a six-month period; contact took the form of calls, texts, emails, or in-person visits, depending on the participant’s preference. Peer recovery coaching services involved immediate access to a personal coach, a local recovery center, and assistance to off-site intervention and recovery resources in the community. During each contact, peer recovery coaches engaged in motivational interviewing and offered emotional, social, informational, and instrumental support customized to each participant’s needs.

#### 2.4.2. Standard-of-Care Control

The standard of care involved a social work consult in which the patient was counseled on the risks of continued substance use and given a contact list for local SUD resources, one of which included recovery coaching. The patient was independently responsible for initiating contact with any of the resources on the list. Given that both intervention and control groups were provided access to recovery coaching, either through the list or the in-person visit, participants were not aware of the alternative study condition.

### 2.5. Measures

#### 2.5.1. Baseline Demographics

Demographic measures included age, gender, race/ethnicity, level of education, employment status, and self-reported duration of substance use. A modified version of the 10-item Drug Abuse Screening Test (DAST-10) was also administered to provide an index of SUD severity [32]. The modification entailed replacing the word ‘drugs’ in the original DAST-10 with the phrase ‘drugs or alcohol’.

#### 2.5.2. Descriptive Measures

At both baseline and six months post-baseline, participants were asked to describe at least one thing that they thought was making it difficult for them to start or maintain recovery from alcohol or drug use. At six months post-baseline, participants were asked the additional two open-ended questions: (1) ‘What would you say is the greatest barrier to your recovery that is outside your control?’ and (2) ‘What is one barrier that you have overcome or made improvement in over the last six months?’.

### 2.6. Procedure

This study was approved by the Institutional Review Board at Prisma Health (IRB Number: 1852883-3) before procedures were implemented. Procedures performed in studies involving human participants were in accordance with the ethical standards of the institutional and/or national research committee and with the 1964 Helsinki Declaration and its later amendments or comparable ethical standards. Baseline data collection occurred from July 2019 to 2 March 2020 (all pre-pandemic). Six-month follow-up assessments occurred from January 2020 to 30 September 2020. Patients were identified as having a SUD through daily chart review. Identified patients were approached by the study physician or a trained medical student in person to inform them about this study and, if interested in participating, were screened for study eligibility. All individual eligible patients included in this study provided written informed consent and were randomized with allocation concealment using REDCap software version 8.10.7 to either a peer recovery coaching intervention or standard-of-care control condition. If randomized to the standard of care, the researcher who administered informed consent (study physician or medical student) placed a social work consult so the participant could receive the SUD resource list. If randomized to the intervention, the researcher contacted a study peer recovery coach to arrange a bedside visit. Intervention participants were informed that information disclosed to their recovery coach would not be shared with study staff and vice versa.

Following randomization, a study researcher first asked participants to verbally respond to the demographic questions and survey measures and then asked participants the open-ended qualitative question. Study researchers were medical students and paid research assistants who were trained in the research procedure. There were no other individuals present besides the patient and the researcher. Given the sensitive nature of the sample, the researcher wrote the participants’ responses down verbatim and then read them back to them to ensure accuracy instead of audio or video recordings.

At six months post-baseline, participants were contacted to complete the six-month assessment. All participants had the option to complete the open-ended questions via email or phone; this procedure was unaffected by the pandemic as it was implemented pre-pandemic. Of the 15 participants who were eligible to complete their six-month assessment before the pandemic, 5 successfully completed the assessment, and 10 were lost to follow-up. Forty-three participants completed the six-month assessment after the onset of the pandemic. Email responses were written by the participant; phone responses were written by the researcher, and the researcher repeated the responses back to the participant to ensure accuracy. Additionally, 3 of the 48 post-intervention respondents completed the questions via email; the remaining 45 completed the questions via phone. Participants were compensated $20 for completing the baseline assessment and $20 for completing the six-month follow-up. Participants who did not respond initially were contacted weekly for two months to complete the six-month follow-up. All data were stored using REDCap software [33].

### 2.7. Coding and Categorization

Open-ended responses were coded using content analysis. The category themes for responses were identified a priori using the ten recovery capital domains delineated in past research [8]: physical health, psychological health, citizenship/community involvement, social support, meaningful activities, environment and safety, coping and life functioning, substance use and sobriety, risk-taking, and recovery experience. These domains represent the ten categories that responses were coded into. Three coders who were not involved in study consent or data collection independently coded all descriptive responses into the ten recovery capital domains; coders were blind to study conditions. Responses could be coded into more than one domain. After each coder completed categorizing the responses into the ten recovery capital domain categories, inter-rater reliability (IRR) Fleiss’ kappa statistics were computed for each open-ended question. Thereafter, the coders met to resolve any disagreements in categorization.

### 2.8. Data Analysis

A Chi-square test was used to compare whether the proportion of participants in the intervention versus control differed in the seven post-baseline recovery domain measures.

To examine changes in difficulties in recovery domains between groups over time, separate generalized mixed-effect models (GLMM) were performed for each recovery capital domain with condition (intervention vs. control) and time (baseline vs. six-month post-baseline) as the predictors, controlling for education. 

## 3. Results

### 3.1. Participant Characteristics

**Figure 1:**
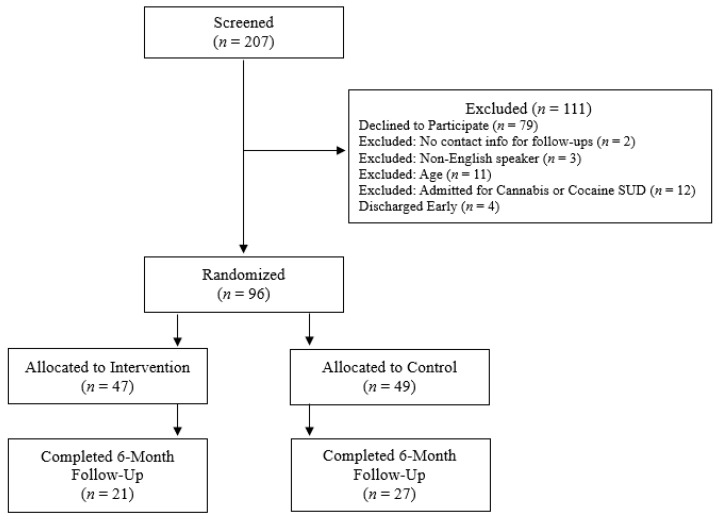
Participant flow diagram for the study.

Ninety-six participants (M_age_ = 40.79, SD_age_ = 9.86, 39 females) consented to this study and were randomized to the intervention (*n* = 47) or control (*n* = 49) condition (Figure 1). Full participant characteristics are shown in Table 1. A total of 21 participants in the intervention condition (M_age_ = 40.48, SD_age_ = 10.16, 10 females) and 27 control participants (M_age_ = 42.04, SD_age_ = 11.00, 13 females) completed the six-month follow-up assessments. The three most common primary diagnosis categories at the time of consent were alcohol-related disorders (19.7%), septicemia (14.0%), and suicidal ideation (5.7%). Only two control study participants established care with a peer recovery coach. All intervention participants established care with a peer recovery coach by study design, and 77.9% remained in contact with their coach at least once every other month throughout the six-month study period.

### 3.2. Coding Results

Fleiss’ *k* for each of the four coding responses ranged from 0.67 to 0.85, which indicates substantial agreement among the three coders (baseline difficulties in recovery (k = 0.839, *p* < 0.001), six months post-baseline difficulties in recovery (k = 0.854, *p* < 0.001), six months post-baseline barriers (k = 0.742, *p* < 0.001), six months post-baseline barriers overcome (k = 0.672, *p* < 0.001)).

Three of the recovery capital domains—citizen/community involvement, risk-taking, and recovery experience—had few participants (≤3 per question) who were coded as having that category as a barrier to their recovery or as a barrier they had overcome by six months post-baseline. Therefore, those three categories were removed from subsequent analyses. Table 2 shows the category, category description, and example of participants’ responses for the recovery capital domain categories that were included in the final analyses.

Frequency results of baseline responses showed that participants reported the greatest difficulties in the environment and safety (28.1%), psychological health (27.1%), and social support (22.9%) domains.

### 3.3. Differences in Six-Month Follow-Up Barriers

At six months post-baseline, participants reported that lack of social support (31.3%) and environment and safety challenges (18.8%) represented the greatest barriers to recovery that were outside their control. There were no significant differences between the intervention and control groups for any of the seven recovery domains (*p* > 0.55). Education level was associated with a higher frequency of psychological health as a recovery barrier (*p* = 0.02) but did not affect any other domains. Table 3 shows these results.

The two domains that participants reported making improvements in at six months post-baseline were substance use and sobriety (52.1%) and coping and life functioning (35.4%) domains. The results of the Chi-square test showed significant differences in the physical health and coping/life functioning recovery domains (Table 4). A greater proportion of participants in the intervention condition reported improvements in physical health (14.3% intervention vs. 0% control, *p* = 0.04) and coping/life functioning (52.4% intervention vs. 22.2% control, *p* = 0.03) compared to the control condition.

## 4. Discussion

One aim of this study was to characterize barriers to SUD recovery using patient input. Using recovery capital as a framework for conceptualizing responses, seven key barriers in recovery capital domains emerged: (1) psychological health difficulties, (2) physical health challenges, (3) lack of social support, (4) insufficient treatment or recovery support to maintain sobriety, (5) environmental and housing concerns, (6) deficits in coping skills, and (7) lack of meaningful activities. An examination of content analysis frequencies indicated that the most prevalent recovery barriers reported at the time of hospitalization were a lack of housing and environmental safety, psychological health problems, and a lack of social support.

Environments with high accessibility to alcohol and illicit drugs can facilitate the acquisition of substances and promote cues that trigger cravings, which can negatively impact someone in their recovery stage [34]. In terms of psychological health barriers, past work suggests that nearly half of individuals with a SUD have a comorbid mental health condition [35]. Moreover, this comorbidity is predictive of heightened SUD severity [36]. These barriers at baseline are, therefore, in line with past findings and show that deficits in specific recovery capital domains may hinder the ability to start the recovery process among hospitalized patients with SUD.

A second aim of this study was to understand patients’ perceived improvements in overcoming recovery barriers six months after experiencing an acute hospitalization from SUD complications. Improvements in recovery support and treatment were overwhelmingly the most common responses across all participants, with over half (52.1%) describing progress in this domain. The second most common theme was in the domain of coping skills, with over a third (35.4%) of participants describing improvement in this area. The qualitative descriptions at six months show a notable shift from responses at baseline during hospitalization. Barriers to recovery support and treatment and coping skills were among the least common themes reported at baseline, yet these two themes were the most prominent six months later. This trend suggests that hospitalization may prompt a shift in patients’ perspective on substance use and trigger readiness to change. Participants in this study underwent both hospitalization due to SUD complications and discussion with study personnel about substance use. This experience may prompt a wake-up call that one’s SUD is severe and that there is a strong need to engage in recovery support services and/or treatment. The difference in the nature of the qualitative responses at the time of hospitalization compared to the six-month follow-up supports this notion. Moreover, these qualitative responses are consistent with prior research demonstrating that hospitalization is a teachable moment to initiate actionable steps toward SUD recovery [18].

Given that this population was experiencing medical complications from SUD, it is somewhat surprising that physical health was not one of the most prominent perceived barriers to recovery. Indeed, less than only 4–6% of participants at both baseline and six-month follow-up reported physical health as a recovery barrier. It is possible that other barriers may be more salient or immediately concerning for those with SUD. Alternatively, it is possible that physical health issues that participants reported may have been adequately addressed during one’s hospitalization rather than becoming an ongoing, chronic problem.

As an exploratory aim, we sought to compare differences in barriers at six-month follow-up between participants involved in a peer recovery coaching intervention compared to the standard of care. Qualitative analysis suggested differences in two key recovery capital domains: coping/life functioning and physical health. However, we note that the frequency comparison for physical health was extremely small (*n* = 3), and thus, the results for physical health should be interpreted with caution. The difference in the coping/life functioning domain suggests that long-term, hospital-initiated peer recovery coaching services may be particularly beneficial in reducing barriers and guiding patients to improve their coping skills. A strength of peer recovery coaching is leveraging past experience to connect with and guide a person seeking recovery. This experience can help instill a sense of positive self-disclosure, role-modeling of self-care, and hope-building [37,38], which supports the observed improvement in coping/life functioning.

### Limitations and Future Directions

Several limitations should be considered when generalizing the study findings. First, one limitation was that this study was terminated early due to the onset of the COVID-19 pandemic in March 2020. The pandemic may also have contributed to attrition rates that were higher than anticipated; there was a 50% attrition rate from baseline to six-month follow-up. This high attrition makes it difficult to draw conclusive inferences from the frequency analyses, as it is possible that differences in frequencies between conditions may be due to follow-up bias rather than differences due to the intervention.

We also note that the relationship between the participant and the peer recovery coach was not measured because of researcher blinding concerns and the need to standardize the research procedure for all participants, regardless of condition. Given the unique roles and responsibilities of a peer recovery coach, we presume that peer recovery coaches provided empathy and adapted to participants’ needs, but this was not empirically assessed in this study.

A further limitation of this study was the conversion of participant responses to open-ended questions into barrier/non-barrier categories. Although rigorous questioning and categorization criteria were imposed to attempt to account for variations in participant responses and researcher interpretation, these variations cannot be entirely eliminated. Therefore, the absence of a particular barrier in a participant’s response may not indicate the absence of that barrier in their experience, but rather, it may be a result of differences in the interpretation of the question or what they are willing to share in their response. We also note that one caveat in qualitative assessment was that all pre-intervention responses were conducted in person at the participants’ bedside, while all post-intervention responses were by phone or email. It is possible that participants may have provided richer, more detailed explanations if the follow-up assessments were conducted in person.

Future studies are warranted to further develop the understanding of patients’ perceived barriers and the effectiveness of peer recovery coaching in mitigating such barriers. It would be worthwhile to conduct mixed-method studies that combined such qualitative measures with quantitative recovery outcomes such as substance use frequency and biopsychosocial functioning in future studies. Finally, achieving recovery from SUD is a long process that often takes years before achieving sustained recovery. While this study explored the effects of a peer recovery coaching intervention on changes in perceived barriers to recovery capital after a 6-month follow-up, a longer follow-up period spanning several years would be informative in assessing the long-term effect of peer recovery coaching on recovery barriers.

## 5. Conclusions

Using descriptive patient input, this study demonstrates that these patients hospitalized with medical complications from SUD report major deficits in recovery capital in environment and safety, psychological health, and social support domains. Six months post-hospitalization, participants report improvement in treatment and recovery services and treatment to support their sobriety as well as improvement in coping skills. Improvement in coping skills was even more pronounced among patients receiving a hospital-initiated peer recovery coaching service. Hospitalization may prompt a call to change that motivates patients to make progress toward SUD recovery. The improvements in these recovery capital domains may be unique to patients actively experiencing SUD-related hospitalization who have moderate-to-severe, long-term SUD.

## Figures and Tables

**Table 1:** Demographics and participant characteristics at baseline for the overall sample and by condition.

Demographic Variables	Overall Sample (*n* = 96)	Intervention Condition (*n* = 47)	Control Condition (*n* = 49)	*p*-Value
	M (SD)	M (SD)	M (SD)	
Age	40.79 (9.86)	39.66 (10.06)	41.88 (9.63)	*p* = 0.27
Years of Education	12.06 (2.00)	11.57 (1.72)	12.53 (2.15)	*p* = 0.02
Years of SUD	13.87 (9.70)	12.59 (9.81)	15.08 (9.54)	*p* = 0.21
	%	%	%	
Gender	40.6% Female, 59.4% Male	38.3% Female, 61.7% Male	42.9% Female, 57.7% Male	*p* = 0.65
Race/Ethnicity				
Black/African American	13.6%	12.8%	14.3%	*p* = 0.83
White/Caucasian	82.3%	83.0%	81.6%	*p* = 0.87
Hispanic/Latino	3.1%	2.1%	4.1%	-
American Indian	1.0%	2.1%	0.0%	-
Employment Status				
Full-time	22.9%	19.1%	26.5%	*p* = 0.39
Part-time	6.3%	6.4%	6.1%	*p* = 0.96
Unemployed	45.8%	46.8%	44.9%	*p* = 0.85
Disabled	22.9%	25.3%	20.4%	*p* = 0.55
Other	2.1%	2.1%	2.0%	-
DAST-10 Severity				
Low	16.7%	12.8%	20.4%	*p* = 0.32
Moderate	12.5%	14.9%	10.2%	*p* = 0.49
Substantial/Severe	45.8%	42.6%	51.0%	*p* = 0.41
Severe	25.0%	29.8%	18.4%	*p* = 0.19
Type of SUD				
Alcohol	50.0%	44.7%	55.1%	*p* = 0.31
Opioids	31.3%	36.2%	26.5%	*p* = 0.31
Methamphetamine	40.6%	46.8%	34.7%	*p* = 0.23

Other denotes retired or homemaker; *p*-values are unavailable for cells smaller than 5; SUD refers to substance use disorder; DAST-10 refers to the Drug Abuse Screening Test.

**Table 2:** Category, category description, and examples of participant responses for the analyzed recovery capital domains.

Category Content	Description	Example Responses [Participant Number]
**Psychological Health** Frequency: 27.1% (26)	Mental health, stress, well-being	“less stress; lots of high-stress events happened over the last year that has escalated drug use” (52)
		“not getting help for emotional issues” (58)
		“self condemnation” (65)
		“I am always stressing about money and it makes me want to do drugs to help cope with the stress of life” (17)
		“Fear and anxiety and a little bit of depression” (21)
**Physical Health** Frequency: 5.2% (5)	Physical health, disabilities, sleep concerns, pain management	“not gaining my strength back; I lost everything out of my body” (51)
		“lack of pain management” (53)
		“being in chronic pain 24/7” (89)
		“my speech pattern has changed and it frustrates me” (73)
**Social Support** Frequency: 22.9% (22)	Relationships and support from family and friends	“need to talk to people when I am having problems” (4)
		“not staying away from certain people and certain friend groups” (96)
		“not changing friends or getting rid of toxic people” (64)
		“no stability in relationships; not having someone to love and who loves equally back; no friends who stay in touch consistently” (35)
		“not having a support system, or someone to hold me accountable for my actions” (65)
**Meaningful Activities** Frequency: 5.2% (5)	Work, hobbies, being active in activities	“no substitute for opiates in terms of goals/activities” (9)
		“haven’t been able to work or engage in my hobbies” (4)
		“not being able to work” (58)
		“not having a full time job to go to and keep my mind busy and active” (91)
		“too much free time” (12)
		“using alcohol to relax at the end of the day is part of my routine, so replacing alcohol with something positive in my daily routine would help. I just don’t have anything else to do” (52)
**Environment and Safety** Frequency: 28.1% (27)	Living situation, housing, environment, safety, transportation, finances	“getting away from where I live” (3)
		“alcohol is everywhere which makes it hard for me stay away from it” (10)
		“move to a different neighborhood” (90)
		“having more money to change the situation I’m in” (24)
		“getting driver’s license, transportation to get back to work” (15)
**Coping and Life Functioning** Frequency: 13.5% (13)	Managing triggers, emotion regulation	“have difficulty with emotion regulation and coping” (5)
		“not sticking to the boundaries I set” (30)
		“my coping mechanisms” (45)
		“not being able to handle thing on her own and not being self-sufficient” (95)
		“trouble dealing with cravings” (53)
		“need to learn how to deal with triggers that make me want to drink” (98)
**Substance Use and Sobriety** Frequency: 8.3% (8)	Abstinence, medication for drug use, recovery meetings, rehabilitation	“not going to recovery meetings” (4)
		“not being on the right medication to stop myself from self medicating” (15)
		“need to be in a supportive program” (21)
		“need Suboxone Program maintenance” (29)
		“not going to therapy & meetings” (59)
		“can’t get into a rehab program” (95)

Numbers represent % (*n*). The frequencies represent the percentage of participants who reported a barrier in each category. The numbers in parentheses in the table refer to the participant number.

**Table 3:** Frequency table by condition in response to the question, ‘What is your greatest barrier to recovery that is outside your control?’.

	6 Months Post-Baseline			
Recovery Capital Domain	Total (*n* = 48)	Control (*n* = 27)	Intervention (*n* = 21)	*p*-Value
Psychological Health	12.5% (6)	14.8% (4)	9.5% (2)	*p* = 0.58
Physical Health	4.2% (2)	3.7% (1)	4.8% (1)	*p* = 0.86
Social Support	31.3% (15)	29.6% (8)	33.3% (7)	*p* = 0.79
Meaningful Activities	8.3% (4)	7.4% (2)	9.5% (2)	*p* = 0.80
Environment/Safety	18.8% (9)	18.5% (5)	19.0% (4)	*p* = 0.96
Coping/Life Functioning	10.4% (5)	11.1% (3)	9.5% (2)	*p* = 0.86
Substance Use and Sobriety	8.3% (4)	7.4% (2)	9.5% (2)	*p* = 0.80

Numbers represent % (*n*).

**Table 4:** Frequency table by condition in response to the question, ‘What barrier have you overcome or made improvement in over the last six months?’.

	6 Months Post-Baseline			
Recovery Capital Domain	Total (*n* = 48)	Control (*n* = 27)	Intervention (*n* = 21)	*p*-Value
Psychological Health	8.3% (4)	7.4% (2)	9.5% (2)	*p* = 0.80
Physical Health	6.3% (3)	0.0% (0)	14.3% (3)	*p* = 0.04 *
Social Support	27.1% (13)	25.9% (7)	28.6% (6)	*p* = 0.84
Meaningful Activities	4.2% (2)	3.7% (1)	4.8% (1)	*p* = 0.86
Environment/Safety	6.3% (3)	3.7% (1)	9.5% (2)	*p* = 0.41
Coping/Life Functioning	35.4% (17)	22.2% (6)	52.4% (11)	*p* = 0.03 *
Substance Use and Sobriety	52.1% (25)	59.3% (16)	42.9% (9)	*p* = 0.26

Numbers represent % (*n*). * denotes statistical significance at the *p* < 0.05 level.

## Data Availability

Data are available to qualified researchers upon reasonable request.
